# Dualistic Roles of High Mobility Group Box 1 in Cancer and Inflammation

**DOI:** 10.1002/cam4.71455

**Published:** 2025-12-07

**Authors:** Wen Zeng, Xu Zhang, Yulu Jiang, Yuxiang Luo, Zuao Wang, Xiaohong Du, Leifeng Chen

**Affiliations:** ^1^ Department of Anesthesiology, The Second Affiliated Hospital of Nanchang University Nanchang Jiangxi Province People's Republic of China; ^2^ Department of Obstetrics and Gynecology, The First Affiliated Hospital of Jinzhou Medical University Jinzhou Liaoning Province People's Republic of China; ^3^ Department of Oncology, The Second Affiliated Hospital of Nanchang University Nanchang Jiangxi Province People's Republic of China

**Keywords:** cancer, HMGB1, inflammation, tumor microenvironment

## Abstract

**Background:**

The High Mobility Group Box 1 (HMGB1) protein, a member of the HMG family, plays a crucial role in both cancer progression and inflammatory responses. HMGB1 can act as a damage‐associated molecular pattern (DAMP) to activate immune responses and modulate inflammation. Its dualistic roles in promoting and inhibiting tumor growth, as well as its involvement in DNA repair and drug resistance, make it a key target for understanding and treating cancer and inflammatory diseases.

**Objective:**

This review aims to explore the dualistic roles of HMGB1 in cancer and inflammation, focusing on its pro‐inflammatory and anti‐inflammatory functions in the tumor microenvironment, its impact on DNA damage repair and tumor drug resistance, and its potential as a therapeutic target for cancer and inflammatory diseases.

**Methods:**

We conducted a comprehensive review of the literature on HMGB1, analyzing its structural features, biological functions, and mechanisms of action in various pathological contexts. We also examined the impact of HMGB1 on tumor progression, immune responses, and metabolic reprogramming in cancer cells, as well as its role in inflammatory signaling pathways.

**Results:**

HMGB1 exhibits both oncogenic and tumor‐suppressive effects in cancer. It promotes tumor growth, metastasis, and immune evasion through mechanisms such as shaping the tumor microenvironment, driving metabolic reprogramming, and inducing drug resistance. Conversely, HMGB1 can enhance anti‐tumor immunity by activating dendritic cells and T cells. In inflammation, HMGB1 acts as a DAMP, activating immune responses via receptors like RAGE and TLR4. Its redox state and subcellular localization determine its proinflammatory or anti‐inflammatory functions. Targeting HMGB1 has shown promise in preclinical and clinical studies, with potential applications in anti‐cancer and anti‐inflammatory therapies.

**Conclusion:**

The dualistic roles of HMGB1 in cancer and inflammation highlight its complexity and potential as a therapeutic target. Future research should focus on elucidating the context‐specific mechanisms of HMGB1, developing precision‐targeted therapies to modulate its multifunctional activities, and translating these findings into clinical practice to improve patient outcomes.

**Abbreviations**
BER: base excision repair
CHUD: chromatin unfolding domain
DAMP: damage‐associated molecular pattern
DC: dendritic cell
ECM: extracellular matrix
FRET: fluorescence resonance energy transfer
GPCR: G protein‐coupled receptor
GSC: glioma stem cell
HCC: hepatocellular carcinoma
HMG: High Mobility Group
HMGB: high‐mobility group box
ICH: intracerebral hemorrhage
LPS: lipopolysaccharide
MDSC: myeloid‐derived suppressor cell
NBD: nucleosome‐binding domain
NEC: necrotizing enterocolitis
NER: nucleotide excision repair
NK: natural killer
OXPHOS: oxidative phosphorylation
PAMPs: pathogen‐associated molecular patterns
P‐gp: P‐glycoprotein
PRCs: Polycomb repressive complexes
PRR: pattern recognition receptor
PTMs: post‐translational modifications
TCA: tricarboxylic acid
TLR9: Toll‐like receptor 9

## 1 Introduction

High Mobility Group Box 1 (HMGB1) is a DNA‐binding protein that associates with eukaryotic chromatin. It derives its name from its high electrophoretic mobility during polyacrylamide gel electrophoresis, a property attributed to its unique structural features [1, 2]. The High Mobility Group (HMG) protein family is categorized into three main subfamilies based on molecular weight, sequence characteristics, and functional divergence: HMGA subfamily (formerly HMG‐14/17); HMGB subfamily (formerly HMG‐1/2); and HMGN subfamily (formerly HMG‐I/Y). HMGA proteins bind A/T‐rich DNA via AT‐hook domains, adopt disordered conformations when unbound, and regulate chromatin via gene expression, remodeling, and structural organization [3]. HMGN proteins bind nucleosomes via nucleosome‐binding domain (NBD), unfold chromatin through chromatin unfolding domain (CHUD), and modulate transcription, repair, and differentiation via post‐translational modifications (PTMs) [3].

The HMGB subfamily includes HMGB1‐4, distinguished by HMG‐box domains that bend DNA. HMGB4, a novel HMGB family member lacking an acidic tail, binds cisplatin‐DNA adducts to inhibit repair, enhancing tumor sensitivity to cisplatin. It regulates plant stress responses and animal neurogenesis/spermatogenesis via chromatin interactions [4, 5, 6, 7, 8]. HMGB1/2/3 share 80% homology, with their structural motifs (HMG‐boxes) mediating sequence‐independent DNA bending and the acidic tail facilitating chromatin interactions‌ [9, 10, 11, 12]. Despite structural similarities, HMGB1, HMGB2, and HMGB3 exhibit functional divergence (Table 1), particularly in tissue‐specific expression patterns and disease association.

**TABLE 1:** Role of HMGB family members in carcinogenesis.

Hallmarks	HMGB family	Key findings	References
Angiogenesis	HMGB1	HMGB1 drives EMT and angiogenesis in HCC via the STAT3/miR‐34a/NF‐κB signaling axis.	[13]
		HMGB1 promotes tumor angiogenesis in gastric cancer via an IL‐8‐mediated mechanism.	[14]
		HMGB1 binds to RAGE to activate NF‐κB and then induces angiogenesis	[15]
		HMGB1 promotes angiogenesis by increasing the expression of VEGFR2 in vascular endothelial cells.	[16]
		HMGB1 promotes angiogenesis by regulating the Akt/eNOS signaling pathway and reactive oxygen species (ROS) production in endothelial cells.	[17]
		HMGB1 promotes angiogenesis by binding to the RAGE receptor, activating downstream signaling pathways, and inducing the expression of pro‐angiogenic factors such as VEGF.	[18]
		The disulfide‐linked form of HMGB1 induces VEGF‐A secretion via TLR4, whereas the fully reduced thiol form mediates endothelial cell migration via RAGE, collectively promoting tumor angiogenesis.	[19]
	HMGB2	Elevated HMGB2 expression demonstrates significant positive correlations with both VEGF upregulation and increased MVD in tumor tissues (*p* < 0.05).	[20]
		HMGB2 induces endothelial cell pyroptosis by activating the RAGE/NF‐κB/NLRP3 signaling pathway, thereby inhibiting angiogenesis.	[21]
	HMGB3	Following uptake of nEXOs‐associated HMGB3 by HUVECs, HMGB3 potently enhances endothelial proliferation and tube formation in a dose‐dependent manner.	[22]
Angiogenesis	HMGB3	HMGB3 promotes angiogenesis by regulating FGF and PDGF.	[23]
Metastasis	HMGB1	HMGB1 activates the PI3K/Akt signaling pathway via the TLR2 receptor, thereby inducing the EMT process.	[24]
		HMGB1 activates the Wnt signaling pathway in pancreatic cancer cells, increases the level of p‐GSK 3β, and thereby influences cell proliferation, migration, and the EMT process.	[25]
		HMGB1 regulates the EMT process by interacting with BRG1 and activating the Akt signaling pathway.	[26]
		HMGB1 promotes NSCLC metastasis by directly transcriptionally activating SNAI1 and indirectly regulating its expression via RSF1‐IT2.	[27]
		HMGB1 promotes tumor invasion and metastasis via activation of TLR4 and RAGE signaling pathways.	[28]
		HMGB1 binds to RAGE and mediates EMT via the production of NF‐kB, p65, iNOS, MMP‐9, and phosphorylation of Rac‐1, ERK1/2, and AKT.	[29]
		Secreted HMGB1 targets other stromal cells, whose released factors can cause ECM proteolytic degradation.	[30]
		HMGB1 binds to RAGE and mediates EMT via MMP‐7, phosphor‐NF‐kB, and Snail.	[31]
	HMGB2	HMGB2 may regulate the cell cycle to influence tumor progression and metastasis.	[32]
		HMGB2 induces T cell exhaustion, impairing their ability to effectively recognize and eliminate tumor cells, thereby facilitating tumor metastasis.	[33]
		HMGB2 promotes tumor metastasis by modulating T cell function and regulating metabolic reprogramming.	[34]
Metastasis	HMGB2	HMGB2 interacts with OCT4 within the nucleus to promote the transcription of EMT‐associated genes, thereby enhancing the migration and invasion capabilities of cells.	[35]
		HMGB2 interacts with RAGE to activate the Ras/MAPK and PI3K/Akt signaling pathways, thereby enhancing cellular adhesion, migration, and invasion capabilities.	[36]
	HMGB3	HMGB3 activates TGF‐β and Wnt/β‐catenin signaling pathways, thereby enhancing the migration and invasion capabilities of tumor cells.	[23]
		HMGB3 regulates the expression of EMT‐associated transcription factors such as Snail, Slug, and Twist, thereby enhancing the motility and invasive capability of tumor cells.	[23]
		HMGB3 regulates cell cycle‐associated proteins, impacts cell proliferation, thereby laying the foundation for tumor metastasis.	[37]
		Overexpression of HMGB3 reverses the inhibitory effects of miR‐5195‐3p on proliferation and metastasis in lung adenocarcinoma cells.	[38]
		In CRC, HMGB3 promotes growth and migration by regulating Wnt/β‐catenin pathway via c‐Myc and MMP‐7.	[39]
Drug resistance	HMGB1	HMGB1 participates in the DNA damage repair process, thereby facilitating the repair of chemotherapy‐induced DNA damage in tumor cells.	[40]
		The HMGB1/c‐Myc axis confers PTX resistance to mCRPC cells.	[41]
		HMGB1 promotes Erk1/2 phosphorylation in the downstream MAPK cascade, thereby enhancing autophagy and conferring resistance to Sorafenib.	[42]
Drug resistance	HMGB1	HMGB1 binds to the RAGE signaling axis to accelerate ATP production, thereby providing energy to sustain the survival and proliferation of tumor cells.	[42]
		HMGB1 enhances chemotherapeutic drug resistance in lung cancer cells by promoting autophagy, inhibiting apoptosis, and enhancing cell survival signaling pathways.	[43]
		HMGB1 promotes autophagosome formation by binding to Beclin1, enhances autophagic activity, and inhibits chemotherapy‐induced apoptosis.	[44]
		HMGB1 enhances tamoxifen resistance in breast cancer cells by binding to TLR4 and activating the NF‐κB signaling pathway.	[45]
		HMGB1, released by tumor cells upon forming complexes with genomic DNA, activates the cGAS‐STING pathway in dendritic cells to enhance anti‐tumor immunity.	[46]
		HMGB1 enhances chemotherapeutic drug resistance in bladder cancer cells by promoting their proliferation and migration and inhibiting their apoptosis.	[47]
	HMGB2	HMGB2 binds to cisplatin‐modified DNA and impairs the NER pathway, thereby reducing cellular sensitivity to cisplatin.	[48]
		Overexpression of HMGB2 may promote chemoresistance in gastric cancer cells by suppressing miR‐23b‐3p function.	[49]
		HMGB2 promotes endocrine therapy resistance in tumor cells by regulating DDX18 expression.	[50]
	HMGB3	HMGB3 inhibits tumor cell apoptosis by binding to cisplatin‐DNA adducts and activating the nucleotide excision repair pathway.	[51]

Members of the HMGB family are implicated in pathological progression processes including inflammation, cancer, and immune system disorders [12, 40, 52, 53, 54, 55]. In this review, we specifically examine the roles of HMGB1 in cancer and inflammation due to its dualistic nature demonstrated in the pathological advancement of these conditions.

## 2 Structural Features and Biological Characteristics of HMGB1

**FIGURE 1:**
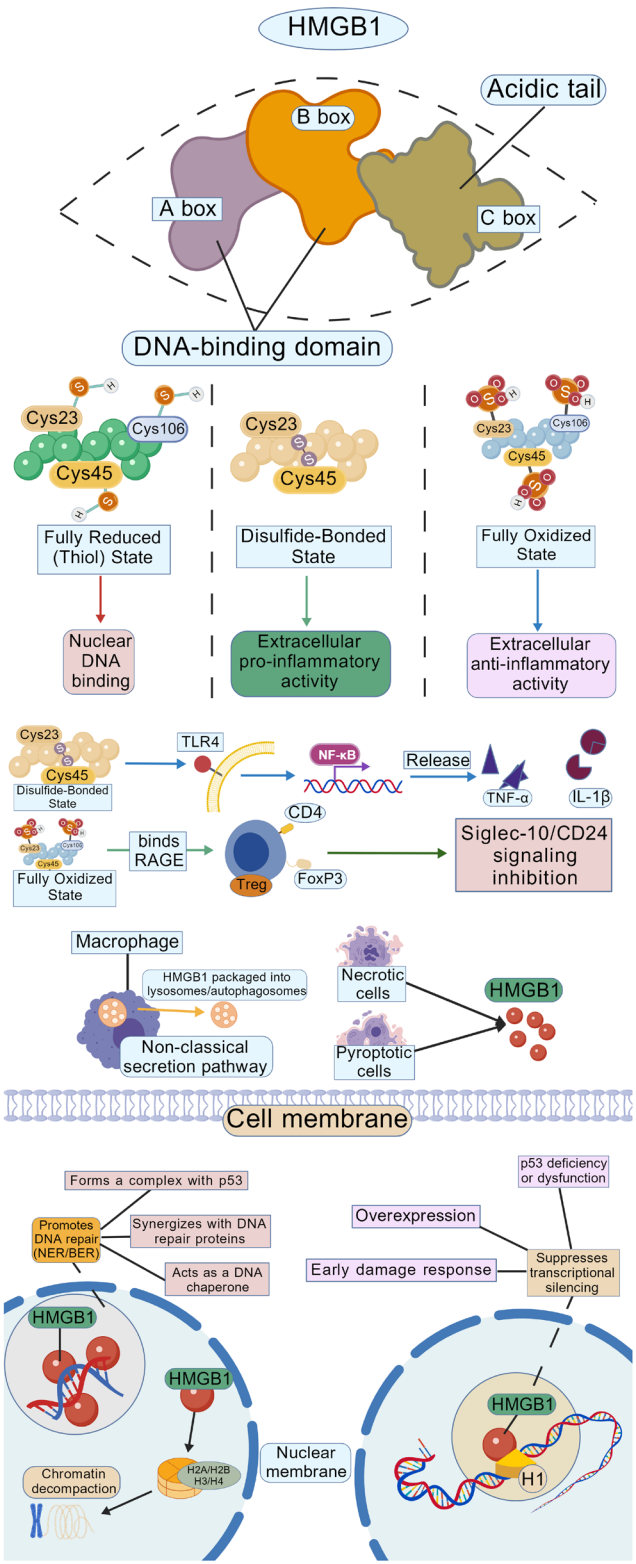
Structure, redox states, and multifunctional roles of HMGB1: The upper half of the figure illustrates the domain architecture of HMGB1, comprising the following regions: the A box (purple), B box (orange), C box (tan), acidic tail (green), and DNA‐binding domains (connecting interdomain regions). The central panel depicts the three distinct redox states of critical cysteine residues (Cys23, Cys45, and Cys106) in HMGB1: Fully reduced state (thiol form): All cysteine residues remain reduced. Function: Nuclear DNA binding. Disulfide bond‐linked state: A disulfide bond forms between Cys23 and Cys45. Function: Extracellular pro‐inflammatory activity. Mechanism: Activates TLR4 and RAGE receptors, promoting the release of inflammatory cytokines (e.g., TNF‐α, IL‐1β) via the NF‐κB signaling pathway. Fully oxidized state: All cysteine residues are oxidized. Function: Extracellular anti‐inflammatory activity. Mechanism: Suppresses immune responses through the Siglec‐10/CD24 signaling axis. The lower half shows that HMGB1 either forms complexes with P53 or coordinates with repair proteins to promote DNA repair, whereas it inhibits transcription under conditions of p53 deficiency or dysfunction, early damage response, and HMGB1 overexpression (created with BioGDP.com).

HMGB1 is the most abundant protein within the HMG family, averaging one HMGB1 molecule per 10–15 nucleosomes in chromatin [11, 56, 57]. HMGB1 exhibits remarkable evolutionary conservation across species, with human and mouse protein sequences sharing 99% amino acid identity. Notably, rat and mouse HMGB1 variants display complete sequence identity at the protein level [56, 58]. HMGB1 contains two homologous L‐shaped DNA‐binding domains (A‐box and B‐box) and an acidic C‐terminal tail. Each domain comprises three α‐helical regions that mediate broad DNA recognition through electrostatic interactions with the DNA backbone. Dynamic conformational flexibility enables HMGB1 to engage DNA‐binding proteins with precision, thereby orchestrating DNA repair, homologous recombination, and transcriptional regulation through chromatin architectural modulation [59]. The A‐box fragment acts as a natural antagonist to HMGB1's pro‐inflammatory B‐box, while recombinant A‐box has been investigated as a therapeutic antagonist [60, 61]. Under physiological conditions, HMGB1 predominantly resides in the nucleus where it binds linear DNA, inducing a helical conformation through electrostatic interactions with the DNA backbone. This structural modulation of DNA alters its affinity for other transcription factors, thereby impacting DNA replication, transcriptional activation, DNA repair mechanisms, and chromatin remodeling processes critical for maintaining genomic stability [62, 63]. HMGB1 additionally interacts with histone H1 to orchestrate chromatin remodeling and transcriptional silencing through its conserved HMG‐box motifs, which stabilize nucleosome‐free regions and recruit Polycomb repressive complexes (PRCs) to epigenetically regulate gene expression [64, 65, 66] (Figure 1). HMGB1 plays a crucial role in DNA repair processes, particularly in the nucleotide excision repair (NER) and base excision repair (BER) pathways [67, 68, 69, 70, 71]. Loss of HMGB1 in cells results in elevated persistent DNA damage post‐repair failure and heightened nuclear instability under stress conditions, triggering aberrant histone release into the extracellular compartment. These histones act as DAMPs that activate Toll‐like receptor 9 (TLR9), thereby amplifying inflammatory cascades through NF‐κB and MAPK signaling pathways. This dysregulation underscores HMGB1's critical role in maintaining nuclear homeostasis through chromatin surveillance mechanisms and epigenetically regulating DAMP release to prevent sterile inflammation [72]. The process by which HMGB1 exits the nucleus and transitions to an extracellular DAMP relies on a series of reversible PTMs. Acetylation serves as a central switch for HMGB1's nuclear‐cytoplasmic translocation: under inflammatory stimuli, histone acetyltransferases (HATs) such as CBP/p300 catalyze acetylation of multiple lysine residues in the NLS1 and NLS2 regions. This neutralizes positive charges, weakens HMGB1's binding to DNA and the nuclear importin karyopherin‐β1, and thereby promotes its dissociation from chromatin and translocation to the cytoplasm [73, 74, 75]. This process is negatively regulated by SIRT1: SIRT1‐mediated deacetylation enhances HMGB1 nuclear retention and suppresses its extracellular release [73, 75]. PARylation primarily functions as an “upstream amplification” mechanism: upon oxidative stress or DNA damage, PARP‐1 is activated and catalyzes PARylation of HMGB1's acidic C‐terminal region. This directly reduces HMGB1's DNA‐binding capacity and amplifies the acetylation signal by recruiting the CBP/p300 complex, synergistically driving nuclear‐cytoplasmic translocation [75, 76]. Phosphorylation acts as a “cytoplasmic retention and secretion trigger”: kinases such as PKCα, CaMKIV, and Akt catalyze phosphorylation at serine/threonine residues. This blocks the re‐binding of NLS to karyopherin‐β1 (preventing HMGB1 re‐entry into the nucleus), enhances HMGB1's interaction with the exportin CRM1, and accelerates nuclear export followed by vesicular secretion [74, 77]. Glycosylation serves as a “structural prerequisite” for HMGB1's efficient secretion: N‐glycosylation at residues N37, N134, and N135 diminishes DNA‐binding affinity, improves CRM1 recognition and protein stability, and protects against ubiquitination‐mediated degradation. Glycosylation‐deficient mutants exhibit nuclear retention and impaired secretion even when acetylated or phosphorylated—confirming glycosylation is a prerequisite for nuclear‐cytoplasmic‐extracellular translocation [75, 78]. Notably, cross‐regulation governs these modifications: PARylation preconfigures nuclear export potential by boosting acetyltransferase activity; phosphorylation and acetylation display mutually exclusive effects at specific sites, finely distinguishing between DNA repair and inflammatory release functional states; and glycosylation indirectly modulates HMGB1's binding interface with RAGE/TLR4 via conformational changes, altering its immune activity as a DAMP [74, 79, 80]. Extracellular HMGB1 exhibits distinct functional repertoires compared to its nuclear counterpart. By binding to receptors such as RAGE, TLR2, and TLR4, HMGB1 activates intracellular signaling pathways, not only amplifying its own secretion in a cascading manner but also regulating the secretion of other inflammatory factors, vascular adhesion molecules, and chemokines, thereby playing a crucial role in the occurrence, development, and maintenance of inflammatory responses and serving as a core component of the inflammatory cytokine network [81, 82, 83, 84, 85].

## 3 The Dual Role of HMGB1 in Cancer

The exact mechanisms by which HMGB1 exerts its effects in cancer remain to be fully elucidated. HMGB1 has been shown to have contradictory effects during the initiation, progression, and treatment of cancer. On one hand, HMGB1 can promote tumor growth, metastasis, and immune evasion. On the other hand, it can also inhibit tumor development under certain circumstances. Its specific role in cancer depends on a variety of factors, including cell type, microenvironment, and the subcellular localization of HMGB1.

## 4 The Oncogenic Effect of HMGB1

**FIGURE 2:**
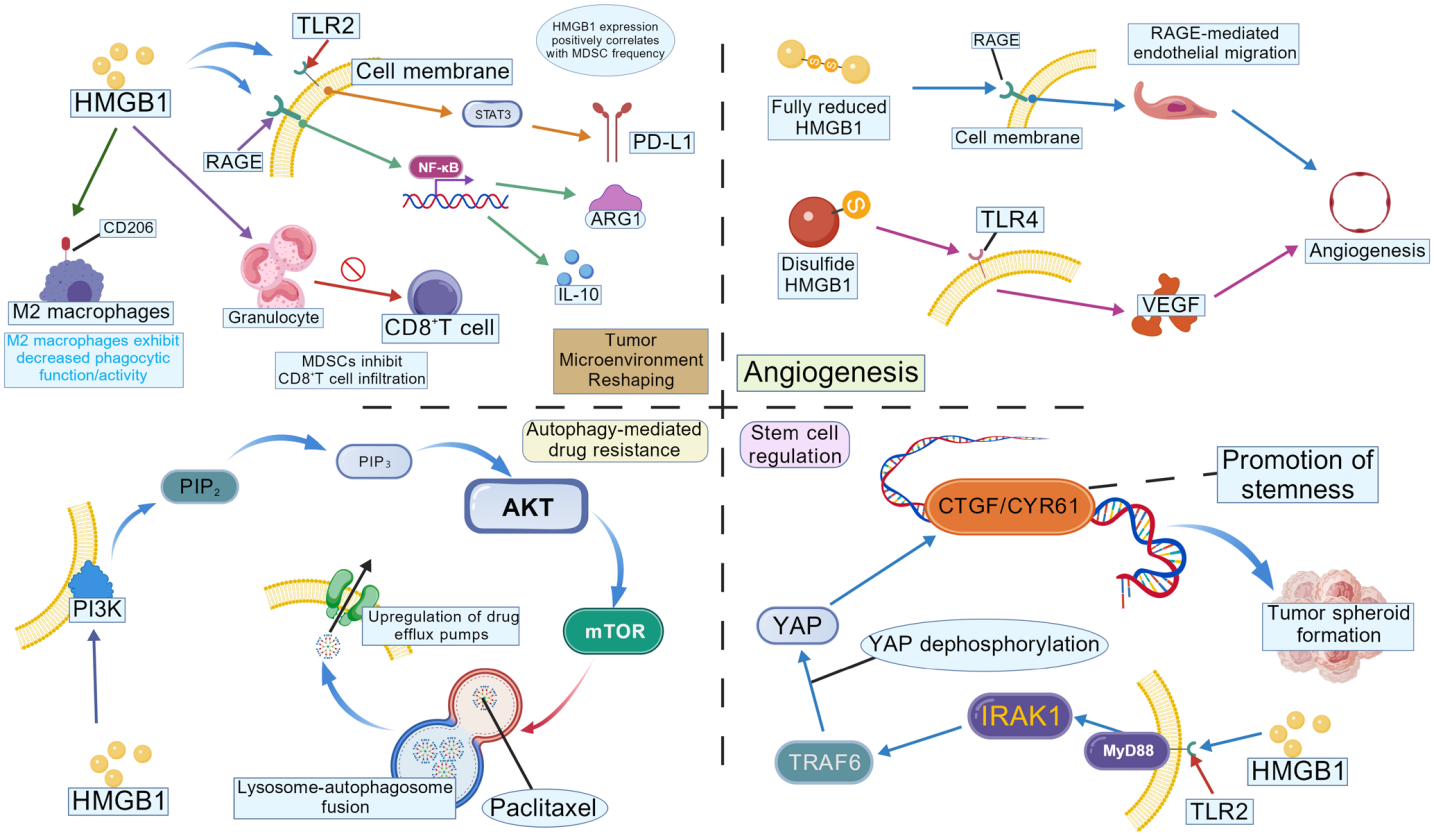
During the extracellular phase, HMGB1 activates downstream NF‐κB and STAT3 signaling pathways by binding to cell membrane surface receptors RAGE and TLR2/4. This interaction drives M2 macrophage polarization (CD206+ ARG1+), granulocyte recruitment, and suppression of CD8+ T cell function, while simultaneously promoting MDSC‐mediated immune evasion. Within the signal transduction network, activation of the PI3K/AKT/mTOR pathway not only induces autophagy‐mediated paclitaxel resistance through enhanced lysosomal‐autophagosome fusion facilitating drug efflux pump activity but also maintains tumor stemness properties via YAP dephosphorylation. Notably, the redox state of HMGB1's disulfide bonds determines functional specificity: the fully reduced form promotes endothelial cell migration via RAGE, whereas the disulfide‐bonded conformation drives VEGF secretion through TLR4 activation to stimulate angiogenesis. This molecular heterogeneity further establishes positive feedback loops through the CTGF/CYR61 and IRAK1‐TRAF6‐MYD88 axes, ultimately enhancing tumor spheroid formation capacity and reinforcing malignant phenotypes (created with BioGDP.com).

Elevated HMGB1 expression correlates with enhanced malignancy, poor prognosis, and chemoresistance across multiple cancer types (Figure 2). Elevated HMGB1 expression correlates with enhanced tumor invasiveness, lymph node metastasis, and shorter overall survival in breast cancer [86, 87, 88]; drives progression and metastasis while associating with chemoresistance in colorectal cancer [89]; And is linked to larger tumor size, lymphatic dissemination, and reduced survival rates in lung cancer [40, 90].

## 5 The Oncogenic Role of HMGB1 in Shaping the Tumor Microenvironment

**FIGURE 3:**
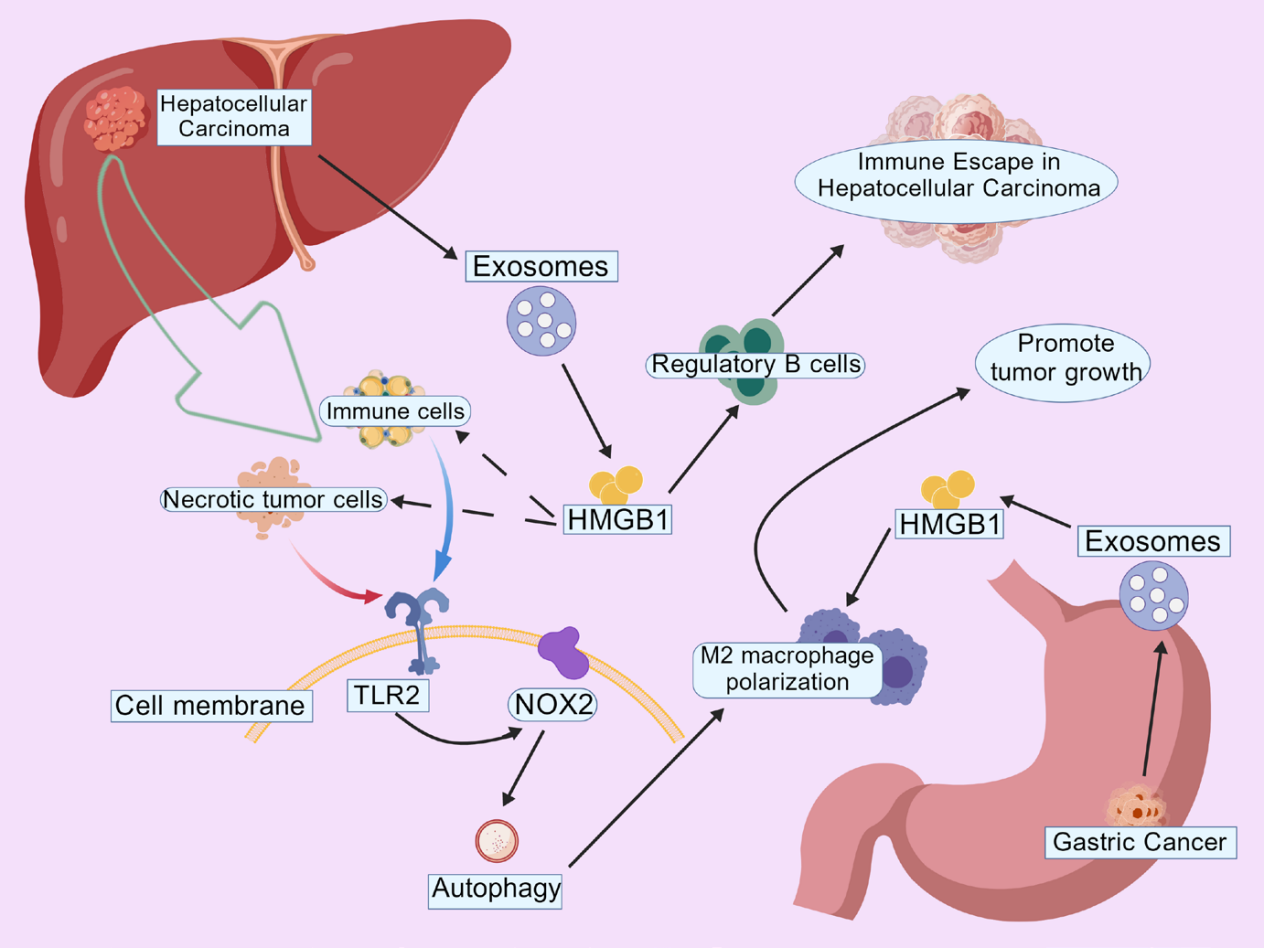
In the hepatocellular carcinoma microenvironment, necrotic tumor cells release HMGB1, which binds to TLR2 on immune cell membranes to activate the downstream NOX2 signaling pathway, thereby inducing autophagy. This autophagic activity further promotes M2 macrophage polarization, establishing an immunosuppressive tumor microenvironment. Tumor‐derived exosomes, as critical mediators, directly participate in immune evasion regulation by delivering bioactive molecules. Concurrently, in gastric tumor regions, analogous regulatory networks mediated by regulatory B cells ultimately elicit tumor‐promoting biological effects (created with BioGDP.com).

HMGB1 activates diverse inflammatory signaling pathways, driving inflammatory cell infiltration and cytokine release to shape a tumor‐promoting microenvironment. HMGB1‐mediated RAGE signaling drives M2 macrophage polarization in the tumor microenvironment, amplifying immunosuppressive activity that fuels tumor growth and progression. Notably, in hepatocellular carcinoma (HCC), HMGB1 drives M2 macrophage polarization via the TLR2/NOX2/autophagy axis, accelerating tumorigenesis [91]. Similarly, HMGB1 in gastric cancer‐derived exosomes induces M2 macrophage polarization to promote tumor growth (Figure 3) [92]. Furthermore, HMGB1 derived from HCC‐derived exosomes enhances immune evasion by expanding TIM‐1+ regulatory B cells [93]. Concurrently, HMGB1 facilitates myeloid‐derived suppressor cell (MDSC) differentiation, recruitment, and activation, amplifying immunosuppression and impairing effector T cell‐mediated cytotoxicity against tumor cells [94]. HMGB1 also exerts a critical oncogenic role by bidirectionally regulating the functional states of DCs and cancer‐associated fibroblasts (CAFs), synergistically shaping an immune‐suppressive and stroma‐remodeling TME. First, HMGB1 binds to TLR4 on DCs, activating the NF‐κB signaling pathway to induce DC maturation, upregulate co‐stimulatory molecule and MHC‐II expression, and enhance antigen‐presenting capacity [95]. However, under chronic high‐dose HMGB1 exposure or specific TLR4 polymorphisms, the HMGB1‐TLR4 signal instead inhibits phagosome‐lysosome fusion, reduces tumor antigen cross‐presentation, and causes failed CD8^+^ T cell priming. It also induces DCs to express Treg‐associated factors, promoting Treg expansion—thereby suppressing CD8^+^ T cell anti‐tumor activity and establishing immune tolerance [96, 97, 98]. Additionally, HMGB1 can activate the cGAS‐STING pathway in DCs via its DNA complex form, transiently inducing type I interferons (IFN‐α/β) to boost CD8^+^ T cell infiltration and stem‐like properties. Yet, without sustained antigen stimulation, this memory population undergoes functional exhaustion, creating an immune “silent” state that facilitates tumor escape [96, 99]. Meanwhile, HMGB1 plays a key role in CAF activation and functional remodeling. In lung adenocarcinoma models, radiotherapy induces tumor cells to release HMGB1, which activates CAFs through the TLR4/PI3K/AKT pathway—driving conversion to the myCAFs phenotype with upregulated FAP, α‐SMA, and Collagen I expression, thereby enhancing tumor fibrosis and invasiveness [100]. In breast cancer, CAFs activate the PI3K/AKT pathway via the G protein‐coupled estrogen receptor (GPR30) to upregulate HMGB1 secretion; HMGB1 then induces tumor cell autophagy through the MEK/ERK pathway in a paracrine manner, strengthening tamoxifen resistance [101]. Furthermore, CAFs actively secrete HMGB1 via an autophagy‐dependent pathway requiring ATG5 and LC3B: HMGB1 is packaged into autophagosomes, transported to the membrane, and released. The extracellular HMGB1 activates TLR4 on tumor cells to induce EMT‐related genes (e.g., Twist, MMP2, MMP9), enhancing migration/invasion. It also reciprocally activates CAF autophagy via autocrine signaling, forming a positive feedback loop that sustains the pro‐tumor CAF phenotype [98, 102]. Additionally, HMGB1 induces CAF secretion of pro‐tumor factors (IL‐6, TGF‐β, VEGF) through a RAGE‐dependent mechanism, promoting tumor angiogenesis, immunosuppressive cell (Treg, MDSC) recruitment, and ECM remodeling—to build a stromal barrier favorable for tumor growth/metastasis [97, 103]. In the TME, the tripartite HMGB1‐DC‐CAF interaction forms a positive feedback immune‐suppressive network: Necrotic foci‐released HMGB1 first induces a “semi‐mature” DC phenotype (high co‐stimulatory molecules but defective IL‐12p70 secretion), generating anergic/regulatory T cells. These tolerant DCs then activate CAF NF‐κB via the OX40L‐OX40 axis, prompting CAFs to secrete more HMGB1/TGF‐β and form an “HMGB1‐tolerant DC‐CAFs” autocrine loop. Concurrently, CAF‐derived lactic acid and collagen remodeling inhibit DC glycolysis/cross‐presentation, reinforcing immune suppression. Ultimately, this leads to cytotoxic T cell exhaustion and upregulation of PD‐L1/CTLA‐4—laying the groundwork for tumor progression/recurrence [99, 104, 105]. In summary, HMGB1 bidirectionally regulates DCs and CAFs via TLR4/RAGE: On one hand, it suppresses DC antigen cross‐presentation and drives tolerance; on the other, it activates CAFs to remodel stromal barriers and sustain HMGB1 secretion, forming a self‐amplifying immune‐suppressive circuit. This synergistically shapes an immune‐suppressive, fibrosis‐enhanced TME, promoting tumor immune evasion, therapy resistance, and metastatic progression.

## 6 HMGB1 Orchestrates Metabolic Reprogramming in Cancer Cells

HMGB1 drives tumor metabolic reprogramming by directly intervening in glycolysis, oxidative phosphorylation (OXPHOS), the tricarboxylic acid (TCA) cycle, and mitochondrial energy homeostasis. In hypoxic glioblastoma, HMGB1 upregulates the HOXD9 → PFKFB3 axis in an autocrine manner, elevates fructose‐2,6‐bisphosphate levels, allosterically activates 6‐phosphofructo‐1‐kinase (PFK‐1), accelerates glucose‐to‐lactate flux, and suppresses mitochondrial oxygen consumption, thereby inducing a “glycolytic lock” phenotype [106]. In colorectal cancer, NK cell‐derived HMGB1 sterically blocks pyruvate‐exporting PKM2 tetramers, forcing tumor cells to abandon OXPHOS and triggering metabolic apoptosis; resistant clones compensate by upregulating the glutamine‐ME1‐NADPH axis to replenish the TCA cycle, achieving metabolic escape [107]. Complementarily, nuclear HMGB1 binds to the SET‐HAT1 complex, reducing H3K9ac/H3K27ac levels at the SASH1 promoter, relieving its transcriptional repression of GLUT1, PFKP, and LDHA, and systemically enhancing glycolytic enzyme density and lactate secretion [108]. At the mitochondrial level, oxidized HMGB1 activates NF‐κB‐p65, inducing bone marrow mesenchymal stem cells to deliver intact mitochondria to cancer cells, boosting OXPHOS capacity and ATP production to sustain self‐renewal of tumor stem cells under glucose deprivation [109]. Conversely, monounsaturated fatty acids mediate HMGB1‐K28 deacetylation via SIRT1, anchoring HMGB1 in the nucleus, reducing extracellular HMGB1‐RAGE‐dependent AKT–mTOR signaling, and downregulating expression of glycolytic rate‐limiting enzymes HK2 and LDHA, reshaping energy metabolism toward fatty acid oxidation [110]. In summary, HMGB1 coordinates key metabolic enzyme activity, substrate utilization, and energy stress responses through a “glycolysis prioritization‐mitochondrial plasticity” dual‐track model, laying the metabolic foundation for tumor proliferation and drug resistance.

## 7 Impact of HMGB1 on Drug Resistance

In early‐stage tumorigenesis, HMGB1‐induced autophagy promotes the selective clearance of damaged organelles and misfolded proteins, thereby preserving intracellular homeostasis to enhance tumor cell survival and proliferative capacity [111]. This cytoprotective autophagy enables tumor cells to maintain viability and proliferate under adverse microenvironmental conditions, such as hypoxia and nutrient deprivation [112, 113, 114]. In the context of tumor therapy, HMGB1‐mediated autophagy promotes chemoresistance in cancer cells. Autophagy diminishes the cytotoxic effects of chemotherapeutic agents by degrading intracellular drug targets or eliminating drug‐induced damaged components, thereby compromising therapeutic efficacy. For instance, elevated HMGB1 expression levels are often observed in chemotherapy‐resistant tumor cells and are positively correlated with enhanced autophagic activity. Inhibition of HMGB1 or autophagy restores sensitivity to chemotherapeutic drugs in these resistant cells [44, 115, 116, 117]. Notably, clinical observations indicate that TLR4 polymorphisms predict response to HMGB1‐mediated therapy resistance, underscoring its relevance to immunogenic cell death and therapy outcomes [118]. Second, HMGB1 reshapes drug efflux and the DNA repair landscape: in cisplatin‐resistant NSCLC cell lines, cytoplasmic HMGB1 colocalizes with P‐glycoprotein (P‐gp) and co‐upregulates MRP and lung resistance‐related protein (LRP) expression, enhancing platinum drug extrusion [40]. Carboplatin‐resistant ovarian cancer models reveal that HMGB1 boosts NER and HRR by recruiting repair factors such as XPA and RAD51—siRNA‐mediated HMGB1 silencing induces accumulated DNA damage, elevates apoptosis, and reduces carboplatin IC_50_ by nearly an order of magnitude [119, 120]. Third, HMGB1 modulates cell death modality switching: in AML, the deacetylase SIRT1 inhibits HMGB1 nuclear‐cytoplasmic shuttling, downregulates ACSL4, and blocks ferroptosis—conversely, SIRT1 deficiency promotes HMGB1 cytoplasmic accumulation, restores ACSL4‐mediated ferroptosis, and reverses cytarabine tolerance [121]. Finally, HMGB1 PTMs form a resistance amplification loop: chemotherapy‐induced, PARP1/SIRT6‐dependent ADP‐ribosylation increases HMGB1 acetylation, accelerates its nuclear export, and sustains autophagy and pro‐survival signaling [122]. Notably, HMGB1 also recruits MDSCs and M2‐TAMs via the RAGE/TLR4‐NF‐κB axis, establishing an immunosuppressive microenvironment that indirectly blunts chemotherapy and immunotherapy efficacy [123, 124]—with immunological details elaborated previously. In summary, HMGB1 systematically drives resistance to platinum agents, anthracyclines, proteasome inhibitors, and targeted therapies in solid and hematological malignancies through a multi‐axis “autophagy‐efflux‐DNA repair‐death inhibition‐epigenetic remodeling” network—providing actionable molecular hubs for reversing therapeutic resistance.

## 8 HMGB1 Promotes Tumor‐Associated Angiogenesis

Tumor angiogenesis serves as a critical mediator of cancer metastasis. Previous studies have demonstrated that HMGB1 plays a pivotal role in tumor angiogenesis across multiple cancer types. Mechanistically, HMGB1 enhances angiogenesis by recruiting and activating macrophages, which subsequently stimulates secretion of VEGF, thereby facilitating tumor vascular formation [15, 125]. HMGB1 has been shown to mediate dual biological functions through its distinct redox states, including the fully thiolated form and the disulfide form: the fully thiolated HMGB1 primarily promotes endothelial cell migration via RAGE‐dependent signaling activation [19]; In contrast, the disulfide form of HMGB1 substantially induces secretion of VEGF‐A via TLR4‐mediated interactions, thereby promoting angiogenesis [16, 19]. Furthermore, HMGB1 directly targets endothelial cells to promote their proliferation and tube formation, while also enhancing angiogenesis by inducing secretion of VEGF‐A [14, 19]. In the tumor microenvironment, HMGB1 creates a permissive microenvironment for angiogenesis by modulating inflammatory responses and extracellular matrix (ECM) dynamics [16]. These findings underscore HMGB1's critical role in tumor‐associated angiogenesis while providing a scientific rationale for developing HMGB1‐targeted antiangiogenic therapies.

## 9 HMGB1 Modulates Cancer Stem Cells (CSCs)

**FIGURE 4:**
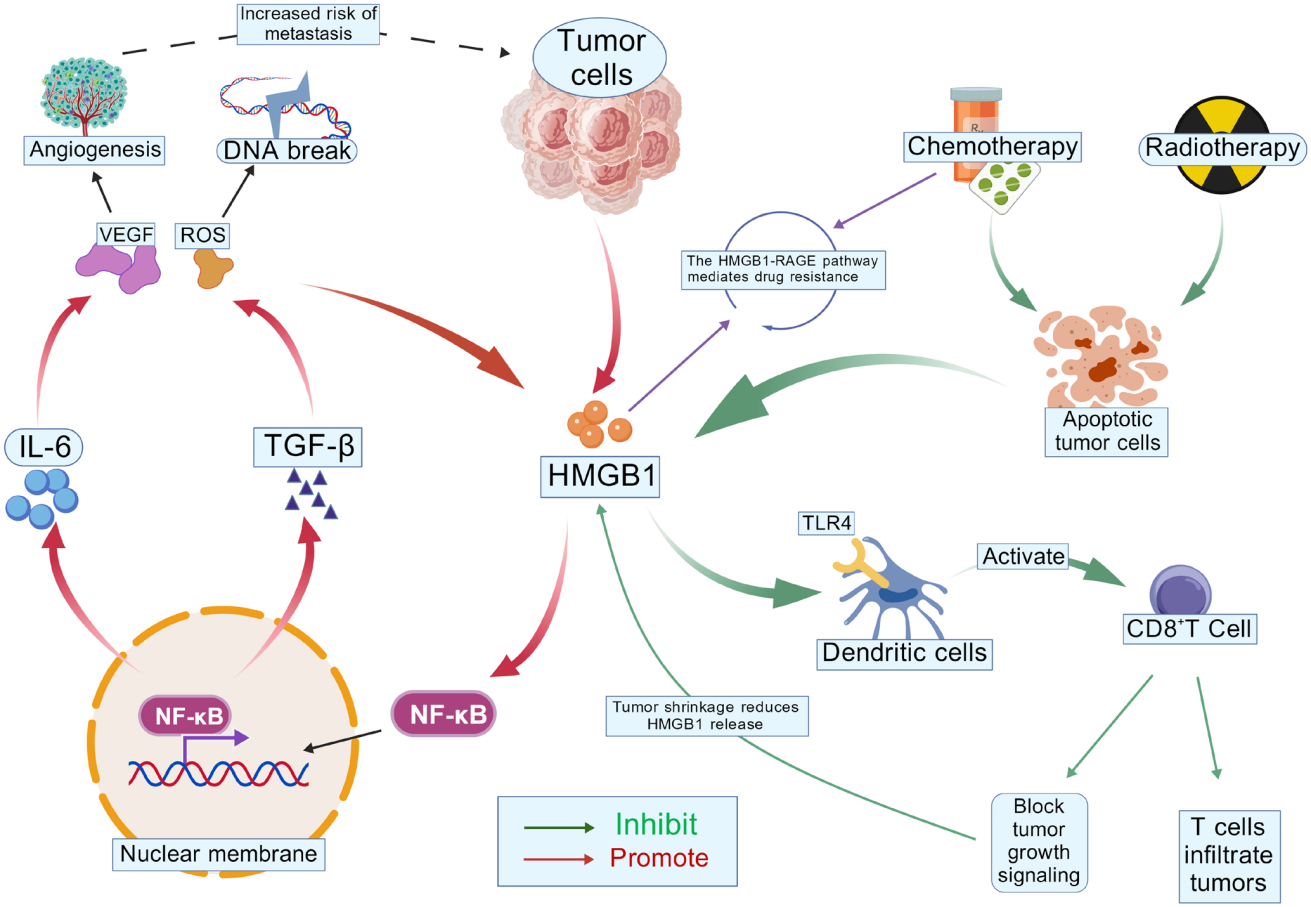
HMGB1 activates the TLR4/NF‐κB signaling axis, inducing NF‐κB nuclear translocation and initiating transcription of pro‐inflammatory cytokines IL‐6 and TGF‐β. These cytokines synergistically promote VEGF‐mediated angiogenesis and ROS‐induced DNA damage, forming a positive feedback loop that drives tumor progression. Further studies reveal that the HMGB1‐RAGE signaling pathway mediates tumor cell chemoresistance via activation of the TGF‐β/SMAD axis, while HMGB1 released from chemotherapy‐induced apoptotic tumor cells exacerbates immunosuppressive microenvironments. Notably, TLR4 receptor‐mediated recognition of HMGB1 by dendritic cells exhibits dual regulatory effects on CD8+ T cell infiltration: it suppresses tumor growth signaling pathways while potentially impairing T cell antitumor function through immune checkpoint molecules (created with BioGDP.com).

HMGB1 modulates the formation, self‐renewal, and CSCs, thereby driving malignant tumor progression. In hematologic malignancies, HMGB1 promotes the formation of CSCs and chemoresistance through activation of inflammatory signaling pathways and modulation of the bone marrow microenvironment [122]. Radiation‐induced HMGB1 release drives tumor cell dedifferentiation into a stem cell phenotype via the TLR2/YAP/HIF‐1α signaling pathway, thereby accelerating tumor recurrence and metastasis [126]. In glioblastoma, HMGB1 drives glioma stem cell (GSC) formation via the TLR2/NEAT1/Wnt signaling axis, which induces chemoresistance, whereas hypoxia‐induced HMGB1 release activates ERK1/2 signaling through RAGE receptor engagement, thereby enhancing the self‐renewal and proliferative capacity of tumor stem cells [127, 128]. Furthermore, in chronic liver disease and HCC, HMGB1 drives hepatocyte dedifferentiation and tumorigenesis via RAGE receptor‐mediated activation of the ERK signaling pathway [129]. In colorectal cancer, APC gene deletion drives Wnt signaling pathway activation, which induces HMGB1 expression and secretion to maintain intestinal stem cells in a crypt progenitor‐like phenotype, thereby providing a cellular basis for tumorigenesis [130]. In breast cancer, HMGB1 enhances tumor stem cell self‐renewal capacity and promotes tumorigenesis and metastasis by activating TLR2, inducing IκBα phosphorylation, stimulating IL‐6 and TGF‐β secretion, and activating STAT3 and Smad3 signaling pathways. Notably, TLR2 inhibition significantly attenuates tumorigenesis and metastasis [131] (Figure 4). Furthermore, in hypoxia‐induced CSCs, HMGB1 forms a complex with p53 to induce bystander cell apoptosis via TLR2/4 signaling, thereby protecting the tumor microenvironment from pathogen‐mediated invasion. This mechanism may provide a novel therapeutic strategy for targeting CSCs [132].

## 10 The Antitumor Effects of HMGB1

**FIGURE 5:**
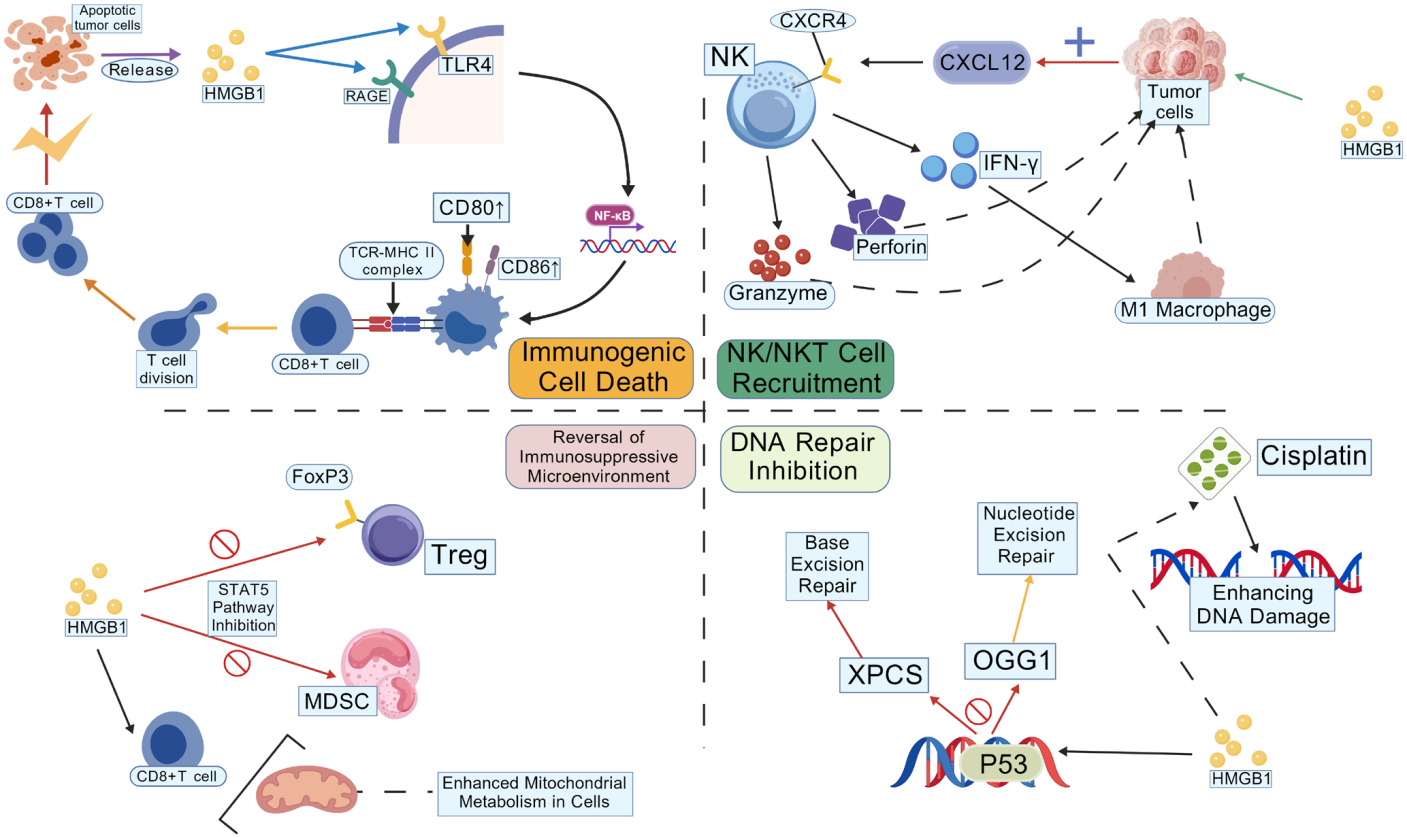
Apoptotic tumor cells release HMGB1, which binds to TLR4 and RAGE on dendritic cell surfaces to activate the NF‐κB signaling pathway, thereby inducing ICD. This process triggers CD8+ T cell proliferation via TCR‐MHC II complex‐mediated activation and enhances effector functions through CD80/CD86 co‐stimulatory molecules. Notably, HMGB1 simultaneously regulates immunosuppressive microenvironments by suppressing regulatory T cell differentiation via the STAT5 signaling pathway while modulating the functional status of MDSCs, consequently dampening CD8+ T cell antitumor activity. In DNA repair mechanisms, HMGB1 enhances tumor cell DNA damage repair capacity by regulating key enzymes XPC and OGG1 in NER and BER pathways. This repair activity establishes a dynamic equilibrium with cisplatin‐induced DNA damage, with the p53 protein exerting critical transcriptional regulation over this process (created with BioGDP.com).

HMGB1 exhibits a complex dual role in tumor progression and anti‐cancer therapy. On one hand, its high expression in tumor cells is frequently associated with malignant progression, including promoting tumor cell proliferation, inhibiting apoptosis, and inducing angiogenesis. Conversely, under specific conditions, HMGB1 may also exert anti‐tumor functions (Figure 5).

HMGB1 enhances anti‐tumor immunity by promoting dendritic cell (DC) maturation and activation via immune stimulation, thereby improving their capacity to efficiently present tumor antigens to T cells [133, 134, 135, 136]. Furthermore, HMGB1 is capable of activating T cells, enhancing immune surveillance, and inducing activation of NK cells and NKT cells, thereby augmenting cytotoxicity against tumor cells [136]. HMGB1 induces immunogenic cell death, activates DCs and T cells to trigger immune responses, and disrupts immune evasion mechanisms in tumor cells, thereby enhancing their susceptibility to recognition and attack by the immune system [137, 138, 139]. In the tumor microenvironment, HMGB1 modulates immune cell composition by depleting immunosuppressive cells (MDSCs, Tregs), increasing immunostimulatory cells (DCs, pDCs), and shifting macrophage polarization, thereby enhancing the anti‐tumor efficacy of the immune system [94, 140, 141]. Furthermore, HMGB1 functions as an immune adjuvant by triggering immune activation via TLR4 binding, thereby potentiating detection and destruction of tumor cells by immune cells [142, 143, 144]. These findings highlight HMGB1's critical regulatory role in anti‐tumor immunity while underscoring the clinical translation potential of research focused on its release mechanisms and therapeutic development [143, 145]. On the other hand, HMGB1 inhibits DNA repair processes through its C‐terminal domain by interacting with DNA repair proteins, thereby enhancing the efficacy of anti‐cancer drugs such as cisplatin. Concurrently, HMGB1 exhibits a synergistic interaction with p53 in jointly regulating DNA repair, with both molecules demonstrating synergistic effects in suppressing DNA repair pathways [146].

## 11 From Bench to Bedside: HMGB1‐Targeted Therapeutic Strategies and Translational Evidence Chain

Extensive research has focused on the role of HMGB1 in tumorigenesis and progression, progressively translating it from laboratory discoveries into actionable clinical intervention strategies and establishing a relatively systematic translational medicine evidence chain. First, at the “mechanism‐intervention‐validation” level, ethyl pyruvate (EP) has emerged as the HMGB1 inhibitor with the greatest current clinical translation potential. Multiple independent studies have confirmed that EP significantly inhibits tumor cell proliferation, migration, and invasion while inducing apoptosis in various solid tumor models, including gastric cancer, malignant mesothelioma, hepatocellular carcinoma, gallbladder cancer, non‐small cell lung cancer, and esophageal squamous cell carcinoma, by blocking the HMGB1–RAGE/TLR4/NF‐κB signaling axis [147, 148, 149, 150, 151]; in diffuse large B‐cell lymphoma, EP also prolongs animal survival by downregulating the HMGB1–Src/ERK pathway [152]. Several agents neutralize HMGB1 directly, including glycyrrhizin and its analogues, which bind HMGB1 and block RAGE/TLR4 signaling, and thrombomodulin, which sequesters HMGB1 through its lectin‐like domain. Both have shown promise in preclinical and clinical settings [153, 154, 155, 156]. Second, targeting the unique immune microenvironment of glioma, recent studies have proposed a “NETs‐HMGB1/RAGE/IL‐8‐CXCR2” positive feedback loop, demonstrating that RAGE inhibitors, CXCR2 antagonists, or PI3K/AKT inhibitors can effectively disrupt this loop [157], providing a theoretical basis for combining HMGB1‐targeted therapy with immunomodulatory strategies. Third, in terms of diagnosis and risk prediction, the dynamic ratio of HMGB1 to anti‐HMGB1 levels has been validated to efficiently distinguish infectious versus autoimmune unexplained fever (AUC = 0.80), offering immediate decision‐making support for clinical choices regarding antibiotic or immunosuppressive therapy initiation [158]; additionally, the association between HMGB1 gene polymorphism (rs1045411) and urothelial cancer susceptibility and pathological invasiveness suggests that this locus could be incorporated into high‐risk population screening and personalized follow‐up systems, enabling more aggressive HMGB1‐targeted interventions for carriers of high‐risk genotypes [159]. HMGB1 has transcended its traditional role as a molecular biomarker, forming a complete translational medicine framework encompassing “risk prediction‐early diagnosis‐therapeutic intervention‐combination potentiation.” Future prospective clinical trials validating the efficacy and safety of its targeted inhibitors in solid tumors and hematological malignancies are expected to provide new, rapidly implementable treatment paradigms for precision oncology.

## 12 The Dual Role of HMGB1 in Inflammation

HMGB1 translocates from the nucleus to the extracellular compartment during cellular stress or injury (including necrosis, apoptosis, and other forms of cell death), where it functions as a DAMP to activate immune responses [160, 161]. HMGB1 is released into the extracellular environment via two distinct mechanisms. Active secretion: Wang et al. first demonstrated that cultured macrophages, upon treatment with lipopolysaccharide (LPS), secrete significant amounts of HMGB1 into the extracellular compartment [162]. HMGB1 lacks a classical signal peptide sequence required for secretion via conventional secretory pathways [163]. Two distinct models have been proposed for the active release of HMGB1 [164]. In the first, HMGB1 is actively secreted into the extracellular space following cellular stimulation [165, 166]. In the second, HMGB1 is packaged into intracellular vesicles (lysosomes or autophagosomes) and subsequently released via vesicular fusion with the plasma membrane [167, 168]. Passive release mechanisms are more heterogeneous, encompassing necrosis, pyroptosis, ferroptosis, oxidative stress, mitochondrial damage, and apoptosis [169, 170, 171]. In addition to redox control, post‐translational modifications (acetylation, phosphorylation, PARylation) are key determinants of HMGB1 secretion [172, 173]. These concerted mechanisms enable massive HMGB1 extracellular release during cellular injury and death, where HMGB1 acts as a DAMP to activate immune responses and amplify inflammatory cascades.

## 13 The Mechanism of HMGB1 in Inflammation

**FIGURE 6:**
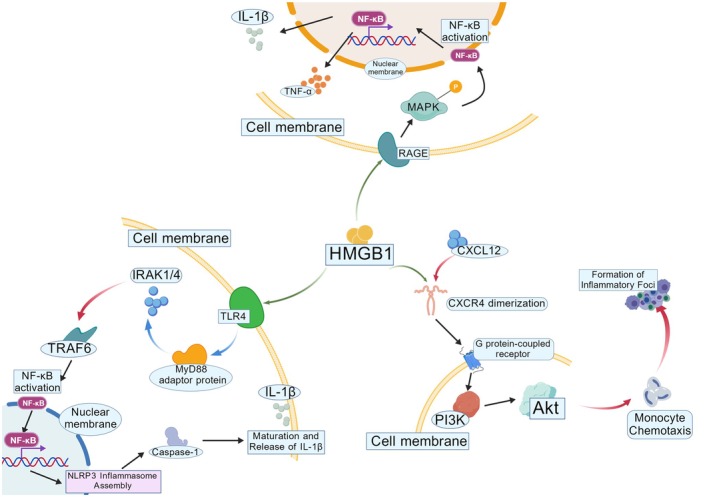
The pattern recognition receptor TLR4 on the cell membrane activates the IRAK1/4‐TRAF6 signaling cascade via the adaptor MyD88, ultimately triggering MAPK phosphorylation and nuclear translocation of NF‐κB. Concurrently, extracellular HMGB1 binding to RAGE stimulates CXCL12‐CXCR4 interaction through activation of the PI3K/Akt pathway, thereby enhancing cellular recruitment within inflammatory microenvironments. Notably, HMGB1 further establishes a positive feedback loop by activating the NLRP3 inflammasome, which mediates caspase‐1‐dependent maturation and release of IL‐1β (created with BioGDP.com).

Extracellular HMGB1 functions as a DAMP, triggering immune activation by binding to receptors such as RAGE, TLR4, and CXCR4. This receptor engagement initiates downstream signaling cascades (MAPK and NF‐κB pathways), stimulating pro‐inflammatory cytokine production (TNF‐α, IL‐1β) (Figure 6).

RAGE has been demonstrated to bind distinct redox forms of HMGB1, thereby mediating divergent biological functions [174, 175]. Following RAGE binding to HMGB1, the MAPK signaling pathway is activated, encompassing three subtypes (ERK1/2, p38, and JNK), which regulate pro‐inflammatory gene transcription in inflammatory contexts [176, 177, 178, 179]. For example, in sepsis and ischemia–reperfusion injury, the HMGB1‐RAGE interaction activates the MAPK pathway (including ERK1/2, p38, and JNK), thereby driving excessive expression of pro‐inflammatory cytokines (e.g., TNF‐α, IL‐1β). Furthermore, HMGB1 acts as a chemokine by binding RAGE, recruiting inflammatory cells to sites of injury and exacerbating inflammation [180, 181]. In neuroinflammation, HMGB1 activates microglia via RAGE binding, triggering the release of pro‐inflammatory cytokines (e.g., TNF‐α, IL‐1β) and thereby exacerbating neuroinflammatory responses [182]. NF‐κB, a master transcription factor, orchestrates the production of pro‐inflammatory cytokines and chemokines. HMGB1‐RAGE binding promotes NF‐κB nuclear translocation, thereby activating inflammatory gene expression [183]. In myocardial infarction and neuroinflammation, the HMGB1‐RAGE interaction drives NF‐κB signaling activation, thereby significantly elevating pro‐inflammatory cytokine levels [184, 185].

TLR4, a widely expressed pattern recognition receptor (PRR), detects diverse exogenous pathogen‐associated molecular patterns (PAMPs) and endogenous DAMPs, thereby driving pro‐inflammatory cytokine production and initiating innate immune responses [186]. J. S. Park experimentally validated the direct interaction between TLR4 and HMGB1 using fluorescence resonance energy transfer (FRET) and co‐immunoprecipitation assays [187]. The binding of HMGB1 to TLR4 activates downstream signaling pathways, including the MyD88‐dependent axis. MyD88, a key adaptor protein, recruits and activates a cascade of kinases and transcription factors, notably NF‐κB. Once activated, NF‐κB translocates to the nucleus, driving transcriptional activation of pro‐inflammatory cytokines (e.g., IL‐1β, TNF‐α, IL‐6) and chemokines, thereby triggering inflammatory responses [188, 189, 190, 191]. In intracerebral hemorrhage (ICH) models, nuclear HMGB1 translocates to the cytoplasm, upregulating TLR4 and MyD88 expression to drive neuroinflammation. Knockdown of HMGB1 reverses the upregulation of inflammatory markers (e.g., IL‐1β, TNF‐α) and ameliorates neuroinflammatory responses [189]. In necrotizing enterocolitis (NEC) models, upregulated HMGB1 and NLRP3 expression exacerbate intestinal inflammation. HMGB1 inhibition alleviates gut inflammation via suppression of the TLR4/NF‐κB signaling pathways, which in turn block NLRP3 inflammasome activation and reduce pro‐inflammatory cytokine (e.g., IL‐1β, TNF‐α) production [190]. The HMGB1/TLR4 interaction represents a pivotal mechanism in inflammatory pathogenesis. By activating downstream signaling pathways, this axis drives pro‐inflammatory cytokine release, amplifying inflammatory responses that accelerate disease progression. While disease‐model‐specific manifestations vary, the HMGB1/TLR4 axis consistently underscores its potential as a therapeutic target across inflammation‐related disorders.

CXCR4, a G protein‐coupled receptor (GPCR) and chemokine receptor type 4, is widely expressed in hematopoietic cells and plays a critical role in leukocyte migration and trafficking [192]. CXCR4 typically requires additional modulators to regulate chemokine‐mediated inflammatory signaling cascades. HMGB1–CXCL12 complex recruits leukocytes through CXCR4; this mechanism was first clarified by Schiraldi et al. [193]. Extracellular thiolated HMGB1 forms a heteromeric complex with CXCL12 (1:2 stoichiometry), subsequently inducing conformational rearrangement of CXCR4 homodimers and retaining them on the plasma membrane via a β‐arrestin 2‐dependent mechanism [194]. The HMGB1‐CXCL12 complex activates CXCR4 receptor‐mediated signaling, driving the recruitment of inflammatory cells (e.g., monocytes and macrophages) to sites of tissue damage—a mechanism particularly critical during the early stages of inflammation [193, 195]. Studies in RSV‐infected nude and BALB/c mouse models demonstrate that HMGB1 recruits NK cells via the CXCL12/CXCR4 axis, thereby driving persistent airway inflammation and hyperresponsiveness. Administration of anti‐HMGB1 neutralizing antibodies alleviates RSV‐induced airway pathology, accompanied by a significant reduction in CXCR4+ NK cells [196]. In a cerulein‐induced acute pancreatitis mouse model, macrophage‐derived HMGB1 exacerbates inflammatory and pain signaling pathways via RAGE and the CXCL12/CXCR4 axis [197]. In summary, HMGB1 forms a complex with CXCL12 to activate the CXCR4 chemokine receptor, thereby driving inflammatory cell recruitment and activation, which plays a pivotal role in inflammation pathogenesis.

## 14 The Anti‐Inflammatory Effects of HMGB1

**FIGURE 7:**
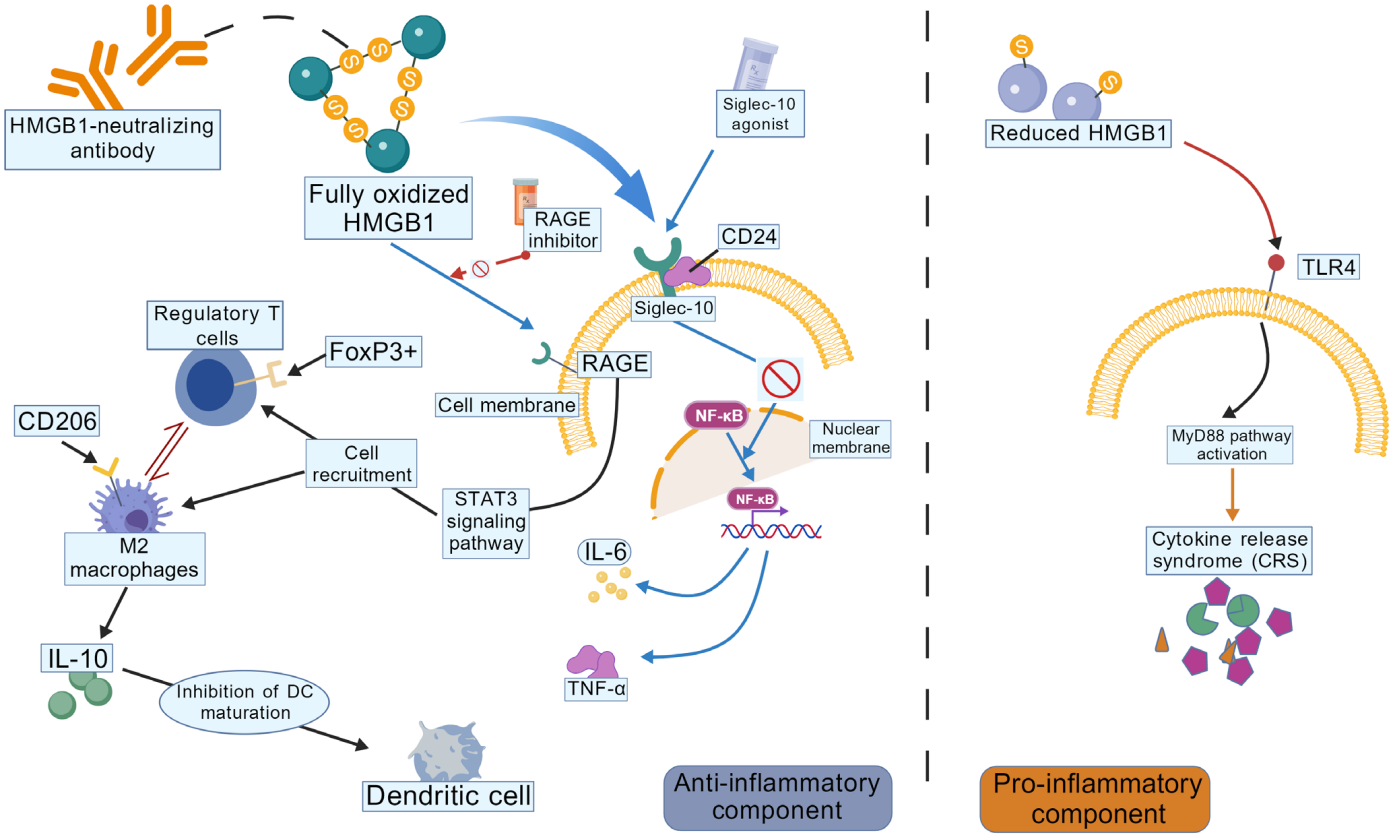
The fully oxidized HMGB1 is specifically recognized and bound by HMGB1‐neutralizing antibodies, thereby blocking its pro‐inflammatory activity. Concurrently, regulatory T cells modulate M2 macrophage differentiation and function via the FoxP3+ regulatory pathway. These M2 macrophages express the CD206 surface marker and secrete anti‐inflammatory cytokines such as IL‐10, effectively inhibiting dendritic cell maturation. Furthermore, M2 macrophages participate in cellular recruitment through the STAT3 signaling pathway, further regulating immune responses. Notably, HMGB1 interactions with cell membrane receptors play pivotal roles in inflammation regulation. Binding of HMGB1 to the RAGE receptor may promote inflammatory responses, which can be blocked by RAGE inhibitors. Simultaneously, HMGB1 can bind to Siglec‐10, which forms a complex with CD24 to inhibit NF‐κB nuclear translocation, thereby reducing pro‐inflammatory cytokine production (e.g., IL‐6 and TNF‐α). In contrast, unmodified HMGB1 binds to TLR4 receptors to activate downstream MyD88 signaling, ultimately driving NF‐κB nuclear translocation and CRS. The diagram distinctly delineates anti‐inflammatory and pro‐inflammatory components, illustrating HMGB1's complex regulatory network in inflammatory microenvironments and its potential as an immunotherapy target (created with BioGDP.com).

HMGB1 contains three cysteine residues (Cys23, Cys45, and Cys106), whose redox states critically dictate its functional activities (Figure 7). HMGB1 exists in three distinct redox states: the fully reduced thiol form, the disulfide‐bonded form, and the fully oxidized form. The fully reduced HMGB1 predominantly localizes in the nucleus, where it mediates DNA binding and chromatin remodeling. In contrast, the disulfide‐bonded HMGB1 exhibits pro‐inflammatory activity, whereas the fully oxidized HMGB1 displays anti‐inflammatory properties [198]. The fully oxidized form of HMGB1 exerts anti‐inflammatory effects by binding to RAGE, which recruits immunoregulatory cells such as regulatory T cells (Tregs), M2 macrophages, and MDSCs. These cells suppress the activity of pro‐inflammatory cells, thereby attenuating inflammatory responses [199]. This dichotomy is consistent with the seminal work of Venereau et al., showing mutually exclusive chemotactic vs. cytokine functions depending on redox status [200]. HMGB1 forms a tripartite complex with CD24 and Siglec‐10, which suppresses NF‐κB activity and thereby reduces pro‐inflammatory cytokine production [201]. HMGB1 plays a pivotal role in anti‐inflammatory responses through its redox‐dependent regulation, receptor interactions (e.g., RAGE), and cooperative binding with other molecules. These mechanistic insights provide novel therapeutic targets for inflammatory diseases.

## 15 HMGB1‐Targeted Therapies in Clinical Translation

Numerous studies have consistently demonstrated that HMGB1, acting as a “late‐phase” inflammatory mediator, drives the inflammatory cascade in diverse pathologies including trauma, sepsis, radiation‐induced injury, autoimmune diseases, and neurodegenerative disorders. Its release from the nucleus to the extracellular space serves as a critical link between acute tissue damage and chronic organ dysfunction. Building on this mechanism, translational medicine research has proposed three categories of interventional strategies: First, non‐specific release inhibitors typified by EP block the HMGB1‐TLR4/NF‐κB positive feedback loop, significantly attenuating post‐traumatic acute lung injury, hemorrhagic shock‐induced hepatic injury, radiation‐induced pulmonary fibrosis, and sepsis‐associated immunosuppression in animal models. These inhibitors have advanced to phase II clinical trials for cardiac surgery, suggesting their potential as resuscitation fluids or adjuvant agents for radiotherapy [202, 203, 204, 205]. Second, high‐affinity HMGB1 antagonists—including recombinant A‐box and glycyrrhizin—competitively bind to RAGE/TLR4, selectively inhibiting pathological inflammation while sparing physiological immunity in models of inflammatory bowel disease (IBD), experimental orchitis, Parkinson's disease, and islet transplantation, thereby exhibiting enhanced safety [206, 207, 208]. Lastly, combination targeting strategies, such as the synergistic use of EP with insulin, calycosin, or glycyrrhizin, have been applied to MODS following burns, acute sinusitis, and the AKI‐CKD transition. These approaches integrate antioxidant and immunomodulatory effects, offering novel avenues for delayed intervention in critically ill patients [209, 210, 211]. Preclinical data further indicate that serum HMGB1 levels may serve as a biomarker to guide personalized anti‐inflammatory treatment: elevated postoperative HMGB1 in children with biliary atresia correlates with reduced 2‐year native liver survival, suggesting potential benefits of glucocorticoids or glycyrrhizin for specific subgroups [212]. In models of tauopathies and systemic lupus erythematosus, HMGB1 inhibition not only attenuates inflammation but also reverses stem cell senescence and cognitive deficits, highlighting its dual potential for organ protection and regenerative repair [213]. Targeted HMGB1 interventions have transitioned from bench to bedside; future efforts should focus on optimizing dose–response relationships, patient stratification, and multicenter trials to validate their safety and efficacy as a broad‐spectrum anti‐inflammatory strategy.

## 16 Conclusion

The dualistic role of HMGB1 in tumor progression and inflammatory responses exemplifies the complexity inherent in cellular physiological and pathological mechanisms. Through comprehensive analysis of its structural features, functional modalities, and context‐dependent roles across diverse microenvironments, we re‐examine the multifaceted nature of this protein in disease pathogenesis. HMGB1 exhibits opposing functions in tumor‐associated contexts, including pro‐inflammatory and anti‐inflammatory effects within the tumor microenvironment, as well as contradictory roles in DNA damage repair and chemoresistance. Its redox status, subcellular localization, and receptor engagement emerge as pivotal determinants of functional diversification. This complexity not only challenges conventional paradigms of disease mechanisms but also illuminates novel avenues for precision medicine and targeted therapeutic interventions.

Future investigations must prioritize elucidating HMGB1's context‐specific functional mechanisms across diverse cellular lineages and tissue microenvironments, while delineating the molecular pathways governing its redox‐dependent regulation. Crucially, future work should integrate translational insights from ongoing HMGB1‐targeting trials and deepen exploration of PTM‐regulated release mechanisms. Furthermore, the development of precision‐targeted therapeutics that differentially modulate HMGB1's multifunctional activities will be critical. Such advancements will not only deepen our understanding of the pathophysiological interplay between tumorigenesis and inflammation but also catalyze the design of tailored therapeutic strategies for HMGB1‐associated pathologies in clinical settings.

## Author Contributions

W.Z., X.Z., and Y.J. conceptualized the article; performed the literature; acquired, analyzed, drafted, and extensively revised the manuscript. Z.W. and Y.L. helped to draw the figures and tables. X.D. and L.C. supervised the study, performed the literature, and reviewed and extensively revised the manuscript.

## Funding

This work was supported through Grants from the National Natural Science Foundation of China (82160364) and the Project of the Jiangxi Provincial Department of Science and Technology (20232BCJ22016).

## Ethics Statement

The authors have nothing to report.

## Consent

The authors have nothing to report.

## Conflicts of Interest

The authors declare no conflicts of interest.

## Data Availability

The authors have nothing to report.
